# Transcription elongation factor AtSPT4-2 positively modulates salt tolerance in *Arabidopsis thaliana*

**DOI:** 10.1186/s12870-023-04060-x

**Published:** 2023-01-23

**Authors:** Ayesha Liaqat, Alamin Alfatih, Sami Ullah Jan, Liangqi Sun, Pingxia Zhao, Chengbin Xiang

**Affiliations:** Division of Life Sciences and Medicine; Division of Molecular & Cell Biophysics, Hefei National Science Center for Physical Sciences at the Microscale; MOE Key Laboratory for Membraneless Organelles and Cellular Dynamics, University of Science and Technology of China, The Innovation Academy of Seed Design, Chinese Academy of Sciences, Hefei, 230027 Anhui Province China

**Keywords:** Salt stress, Salt tolerance, Transcription elongation factor, *AtSPT4-2*, *Arabidopsis thaliana*

## Abstract

**Background:**

Salt stress significantly influences plant growth and reduces crop yield. It is highly anticipated to develop salt-tolerant crops with salt tolerance genes and transgenic technology. Hence, it is critical to identify salt tolerance genes that can be used to improve crop salt tolerance.

**Results:**

We report that the transcription elongation factor suppressor of Ty 4-2 (SPT4-2) is a positive modulator of salt tolerance in *Arabidopsis thaliana*. *AtSPT4-2* expression is induced by salt stress. Knockout mutants of *AtSPT4-2* display a salt-sensitive phenotype, whereas *AtSPT4-2* overexpression lines exhibit enhanced salt tolerance. Comparative transcriptomic analyses revealed that *AtSPT4-2* may orchestrate the expression of genes associated with salt tolerance, including stress-responsive markers, protein kinases and phosphatases, salt-responsive transcription factors and those maintaining ion homeostasis, suggesting that *AtSPT4-2* improves salt tolerance mainly by maintaining ion homeostasis and enhancing stress tolerance.

**Conclusions:**

*AtSPT4-2* positively modulates salt tolerance by maintaining ion homeostasis and regulating stress-responsive genes and serves as a candidate for the improvement of crop salt tolerance.

**Supplementary Information:**

The online version contains supplementary material available at 10.1186/s12870-023-04060-x.

## Background

Plants have evolved sophisticated mechanisms to manage intracellular perturbations arising under various abiotic stresses, especially under salt stress [1–4]. Previous studies have demonstrated that salt-tolerant plants respond to salinity stress either through the initial response encompassing sensing, signaling, and activating the pathways for transporting sodium ions (Na^+^) outside of the cell or through the subsequent lateral response, which comprises detoxification of Na^+^, epigenetic modification of chromatin, and accumulation of organic osmolytes [5]. mRNA splicing and transcriptomic changes are also lateral responses that improve plant adaptation under salt stress [6–8]. It is clearly aphoristic that, in striving to develop salt-tolerant plants, the resultant salt-tolerant plants must be capable of regulating their biochemical and physiological processes to reduce oxidative damage, improve osmotic and ionic homeostasis, adjust cell division patterns, and modulate overall growth under salinity stress [4].

The initial response of plants to salt stress starts as soon as an excessive Na^+^ concentration is perceived. Salt-tolerant plants respond to such initial accumulation of Na^+^ either by actively exporting Na^+^ from their cells through the plasma membrane-bound Na^+^/H^+^ antiporter or by actively importing Na^+^ into the vacuoles through the vacuolar membrane-bound Na^+^/H^+^ antiporter [1, 9]. As a result of this distribution of Na^+^ into vacuoles or away from the cytoplasm, the cytoplasmic Na^+^ concentration is reduced, thus helping plants tolerate salt stress [9]. In salt-sensitive plants, on the other hand, ionic stressors emerge as a result of the accumulation of Na^+^ within cells. For instance, due to the presence of excessive salt (NaCl) in the environment, competition occurs between Na^+^ and potassium ions (K^+^) for access to intracellular spaces, which ultimately leads to K^+^ deficiency [10]. K^+^ is essential for a plethora of cytoplasmic metabolic processes, such as ribosome operations, enzyme reactions, and protein production. However, the accumulation of Na^+^ instead of K^+^ hinders metabolic activities [11]. Therefore, Na^+^ extrusion and/or intracellular compartmentalization are the most effective ways of preventing excessive Na^+^ accumulation in the cytoplasm and are used as the key parameters in the assessment of salt-tolerant plants [12].

Unlike the rapid initial response, the lateral type response to salt stress is triggered tardily after a plant cell confronts higher salts for a prolonged period of time and/or higher amounts of Na^+^ accumulate within the cytoplasm. The accumulation of Na^+^ in the cytoplasm further induces the production of reactive oxygen species (ROS), causes disruptive signal transduction, imbalanced ionic homeostasis, and alters gene expression in saline environments [13–17]. Although the lateral type of response is obtuse compared to the rapid initial response, the lateral type exhibits a more effective and permanent response to salt stress, as it involves more coordinated mechanisms, especially those regulated by transcription factors (TFs). A reason why TFs are targeted in developing transgenic salt-tolerant plants with permanently enhanced salinity tolerance. Transgenic salt-tolerant plants can be developed after the identification of putative genes and TFs that can effectively modulate ionic homeostasis and regulate cellular mechanisms under salinity stress [9]. Several types of ion transporter genes are also targeted in developing salt-tolerant transgenic plants, as they are involved in the mechanisms for lowering Na^+^ toxicity through sequestration of cations into vacuoles or into extracellular spaces and thus can ultimately maintain intracellular ion homeostasis and protect plants from other consequences, such as ROS [18]. For instance, overexpression of *Arabidopsis* tonoplast-located Na^+^/H^+^ antiporter (*AtNHX1*) [19], *Arabidopsis* vacuolar H^+^-pyrophosphatase gene (*AtAVP1*) [20, 21], *Thellungiella halophila* H^+^-pyrophosphatase gene (*TsVP*) [22, 23], plasma membrane-localized sodium/proton antiporter K2-NhaD [24], and coexpression of *TsVP* and *AtNHX1* [25] have demonstrated improved salt tolerance in transgenic plants. Likewise, the overexpression of rice *SNAC1* [26], *Arabidopsis Enhanced Drought Tolerance1/Homeodomain Glabrous11* (*EDT1/HDG11*) [27], and maize *ABRE Binding Protein 9* (*ABP9*) [28] have shown improved salt tolerance in several transgenic plants. Although numerous genes and TFs have been practically exploited in developing transgenic salt-tolerant plants, many important stress-responsive genes and TFs have yet to be exploited for their potential in improving plant stress tolerance [29].

The genomes of natural halophytes allow for reference genetic information in developing other genetically modified salt-tolerant plants. *Thellungiella salsuginea*, synonymously known as *Thellungiella halophila*, is a near relative of *Arabidopsis thaliana* and is referred to as model halophyte due to its natural potential of high salinity tolerance as well as ease of cultivation [30]. Moreover, their short life cycles, self-pollination ability, and relatively small genome make it a model in the study of salt tolerance (Bressan, Zhang et al. 2001, Inan, Zhang et al. 2004, Volkov, Wang et al. 2004). To date, several genes from these halophytes have been identified and analysed to understand their salinity tolerance mechanisms [23].

One of the identified salt tolerance genes from *Thellungiella halophila* is *Salt Tolerance 5* (*ThST5*), which we previously isolated [31] and evaluated in cotton [32]. *ThST5* is a homolog of the *Arabidopsis SPT4-2* gene [33]. In *Arabidopsis*, *SPT4-1* and *SPT4-2* encode the protein SPT4 [34]. This zinc-finger protein associates with another TF called SPT5 via its N-terminal NusG (NGN)-binding domain to form the SPT4/SPT5 complex, which binds to RNA polymerase II to act as a transcription elongation factor [33–35]. RNA interference (RNAi) knockdown mutants of *Arabidopsis* showed slower root growth and fewer lateral roots than the wild type, demonstrating the role of SPT4 in root growth [33, 36]. However, the function of *Arabidopsis* SPT4 in resistance to abiotic stresses has not yet been elucidated.

In the present study, we aimed to analyze the role of *AtSPT4-2* in salt tolerance using *AtSPT4-2* knockout mutants and overexpression lines. Our results clearly showed that overexpression of *AtSPT4-2* improved the salt tolerance of transgenic plants, while the knockout mutants displayed a salt-sensitive phenotype. To probe the underlying mechanisms, we performed transcriptomic analyses and revealed that *AtSPT4-2* positively regulates an array of salt tolerance-associated genes. Together, our results demonstrate that *AtSPT4-2* is a positive modulator of salt tolerance in *Arabidopsis* and may be a promising candidate for the improvement of salt tolerance in crops.

## Results

### *AtSPT4-2* confers salt tolerance at the germination and seedling stages

**Fig. 1:**
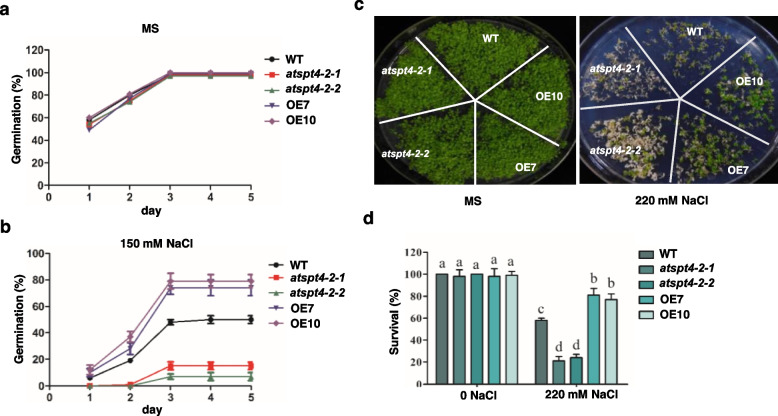
*AtSPT4-2* improves seed germination and seedling survival under salt stress. **a-b** Germination rate. The seeds of wild-type (WT), knockout mutants (*atspt4-2-1* and *atspt4-2-2*), and overexpression lines (OE7 and OE10) were germinated on MS medium without (**a**) or with 150 mM NaCl (**b**) for 5 days, and the germination rate was calculated at the indicated time points. Values are the mean ± SD (*n* = 4 replicates, 40 seeds per replicate). Different letters indicate significant differences by one-way ANOVA (*P* < 0.05). **c-d** Seedling survival. The seeds of WT, *atspt4-2-1*, *atspt4-2-2*, OE7 and OE10 were germinated and grown horizontally on MS medium with or without 220 mM NaCl for 7 days. Photographs were taken (**c**), and the survival rate was calculated at day 7 (**d**). Values are the mean ± SD (*n* = 4 replicates, 40 seeds per replicate)**.** Different letters indicate significant differences by one-way ANOVA (*P* < 0.05)

To investigate the role of *AtSPT4-2* in salt tolerance, we generated two knockout mutants (*atspt4-2-1* and *atspt4-2-2*) via gene editing as well as two overexpression lines (OE7 and OE10) and verified their mutation and expression levels (Fig. S1). We performed seed germination assays with knockout mutants *atspt4-2-1* and *atspt4-2-2* and wild-type (WT) and overexpression lines (OE7 and OE10). On normal MS medium, there was no obvious difference in the germination rate of all genotypes (Fig. 1a). However, when germinated on MS medium with 150 mM NaCl, the seed germination of knockout mutants was more sensitive than WT (with a germination rate of 10% vs. 50%, respectively, at day 5), while the OE lines exhibited a germination rate of nearly 80% at day 5 and were significantly more tolerant than the wild type (Fig. 1b). We then tested the survival rate of all genotypes by germinating and growing on MS medium with or without 220 mM NaCl. On MS medium without NaCl, no difference was shown among the genotypes with a nearly 100% survival rate, whereas on MS medium with 220 mM NaCl, a significant difference in survival rate was seen among the genotypes. The knockout mutants exhibited the lowest survival rate of ~ 20%, while the overexpression lines had the highest survival rate of ~ 80% compared with the wild type with ~ 60% (Fig. 1c, d). These results indicate that *AtSPT4-2* positively affects the germination rate in response to salt stress.

### *AtSPT4-2* positively regulates salt tolerance in soil-grown plants

**Fig. 2:**
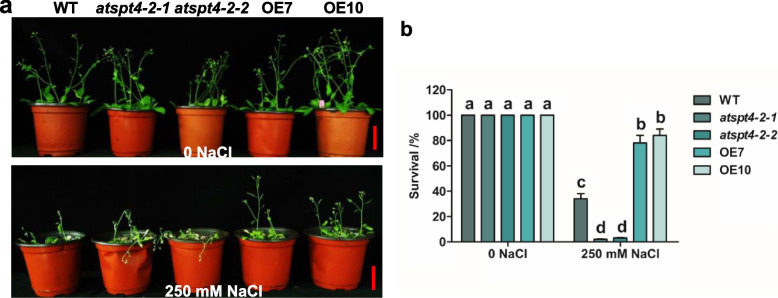
*AtSPT4-2* enhances the salt tolerance of soil-grown plants. **a-b** Seeds of wild-type (WT), knockout mutants (*atspt4-2-1* and *atspt4-2-2*), and overexpression lines (OE7 and OE10) were germinated on normal MS medium and allowed to grow for 7 days on MS medium. The seedlings were transferred to soil pots and grown for 3 weeks. Three-week-old plants were irrigated once with or without 250 mM NaCl, allowed to grow for 1 week, reirrigated once with only water and allowed to grow for another 5 days. After 12 days of treatment, photographs were taken (**a**), and the survival rate was calculated (**b**). Values are the mean ± SD (*n* = 3 replicates, 30 pots per replicate, 6 plants per pot). Different letters indicate significant differences by one-way ANOVA (*P* < 0.05)

To assess the performance of transgenic *Arabidopsis* plants in response to salt stress, a salt tolerance assay was performed on plants grown in soil. Under normal growth conditions, there was no obvious difference in morphology or survival rate among the genotypes (Fig. 2). However, when treated with 250 mM NaCl, the OE lines (both OE7 and OE10) showed a higher survival rate (~ 80%), while the mutants (*atspt4-2-1* and *atspt4-2-2*) exhibited a lower survival rate (< 5%) than the wild type (~ 35%) (Fig. 2b). Taken together, these results demonstrate that *AtSPT4-2* positively regulates salt tolerance in plants grown in soil.

### Expression pattern of *AtSPT4-2* and *AtSPT4-2* is induced by salt stress

**Fig. 3:**
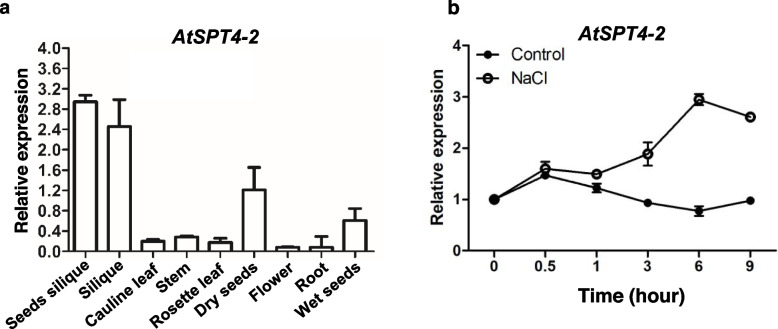
Expression pattern of *AtSPT4-2*. **a** The transcript level of *AtSPT4-2* in different tissues under normal conditions. RNA was extracted from different tissues of 3-week-old wild-type (WT) plants grown in soil, and the transcript level of *AtSPT4-2* was analyzed by qRT–PCR. *Ubiquitin 5* (*UBQ5*) was used as an internal control. Values are the mean ± SD (*n* = 3 replicates). **b ***AtSPT4-2* expression is responsive to NaCl. Seven-day-old WT seedlings grown on normal MS medium were transferred to liquid MS medium containing 0 (control), 150 mM NaCl for 0, 0.5, 1, 3, 6, and 9 hours. Total RNA was isolated from whole seedlings to analyze the transcript level of *AtSPT4-2* by qRT–PCR. *Ubiquitin 5* (*UBQ5*) was used as an internal control. Values are the mean ± SD (*n* = 3 replicates)

To investigate the expression pattern of *AtSPT4-2* in different tissues of *Arabidopsis* plants, we performed qRT–PCR analyses and found that *AtSPT4-2* was expressed in all the tissues examined but at higher levels in siliques and seeds (Fig. 3a) and induced by salt stress compared with that of control (Fig. 3b). These results show that *AtSPT4-2* is induced by salt stress, suggesting that *AtSPT4-2* may play an important role in the salt response.

### Nuclear localization of AtSPT4-2 is responsive to salt stress

**Fig. 4:**
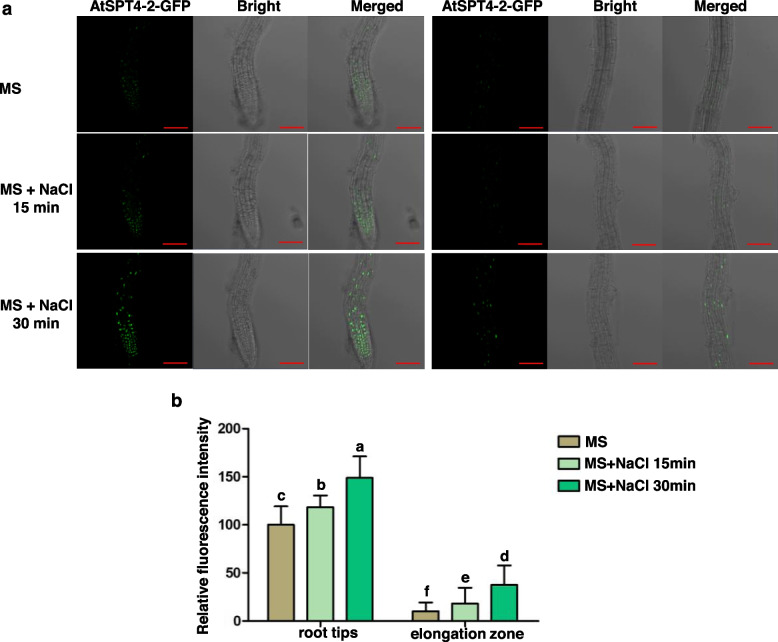
Nuclear localization of AtSPT4-2. **a** Subcellular localization of the AtSPT4-2-GFP fusion protein. *35Spro:AtSPT4-2-GFP* transgenic seedlings were grown on MS medium for 7 days and then transferred to liquid medium supplemented with 150 mM NaCl for 0, 15 and 30 minutes. The green fluorescence of *35Spro:AtSPT4-2-GFP* transgenic root tissue was observed under a ZEISS 880 confocal laser scanning microscope with an excitation of 488 nm and an emission of 525 nm wavelengths. Bar = 20 μm. **b** Relative fluorescence intensity. The relative fluorescence intensity of AtSPT4-2-GFP in Fig. 4a was quantified with ImageJ software (NIH, USA). Values are the mean ± SD (*n* = 10 seedlings). Different letters represent significant differences between samples by one-way ANOVA (*P* < 0.05)

To study the subcellular localization of the AtSPT4-2 protein and the response of AtSPT4-2 accumulation to salt stress, we generated *35Spro*:*AtSPT4-2*-*GFP* transgenic lines. The transgenic plants showed that the SPT4-2-GFP fusion protein was localized to the nucleus in the root tips (Fig. 4a, left) and elongation zone (Fig. 4a, right) under normal conditions on MS medium. When treated with NaCl, GFP signals significantly increased in the nucleus, indicating that SPT4-2-GFP accumulation was significantly increased. Moreover, the relative fluorescence intensity of salt treatment in the root tips and elongation zone was significantly higher than that of the untreated control (Fig. 4b). These results suggest that the SPT4-2-GFP level is enhanced under salt stress, which can be attributed to NaCl-induced transcription of endogenous *AtSPT4-2* and possibly enhanced translation of *AtSPT4-2* mRNA. These results indicate that the accumulation of SPT4-2 protein in the nucleus is increased under NaCl treatment, suggesting that it might play a role in the salt response.

### AtSPT4-2 increases proline content and ROS-scavenging enzyme activities

**Fig. 5:**
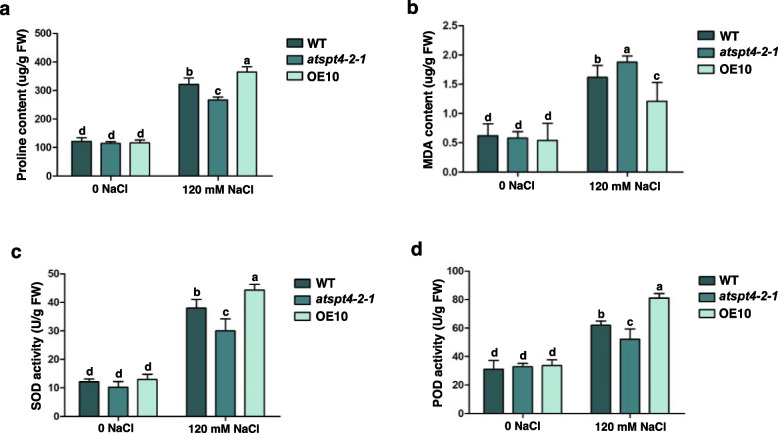
*AtSPT4-2* affects physiological indexes under salt stress. **a-d** The seeds of WT, *atspt4-2-1*, and OE10 were germinated on MS medium without or with 120 mM NaCl for 5 days, and then the proline content (**a**), malondialdehyde content (MDA, **b**), superoxide dismutase activity (SOD, **c**), peroxidase activity (POD, **d**) were quantified. Values are the mean ± SD (*n* = 3 replicates, 120 seeds per replicate). Different letters indicate significant differences by one-way ANOVA (*P* < 0.05)

Salt stress induces the production of reactive oxygen species (ROS) and the accumulation of osmolytes. We further analysed the proline and malondialdehyde (MDA) contents and the activity of antioxidant enzymes, including superoxide dismutase (SOD) and peroxidase (POD), in the WT, *AtSPT4-2-1* and OE10 lines. The proline content, MDA content, and SOD and POD activities were not significantly different among the genotypes in the absence of salt stress but increased under salt stress. The proline level was also higher in the OE10 line than in the WT (Fig. 5a), whereas the MDA content was lower in OE10 and higher in the knockout mutant (Fig. 5b). Moreover, the activities of SOD and POD were significantly increased in OE10 compared with WT but decreased in *the atspt4-2* mutant (Fig. 5c, d). Hence, these results suggest that *AtSPT4-2* could improve the intracellular ROS scavenging ability by increasing the activities of SOD and POD.

### RNA-seq analyses reveal that AtSPT4-2 modulates the expression of salt response-associated genes

**Fig. 6:**
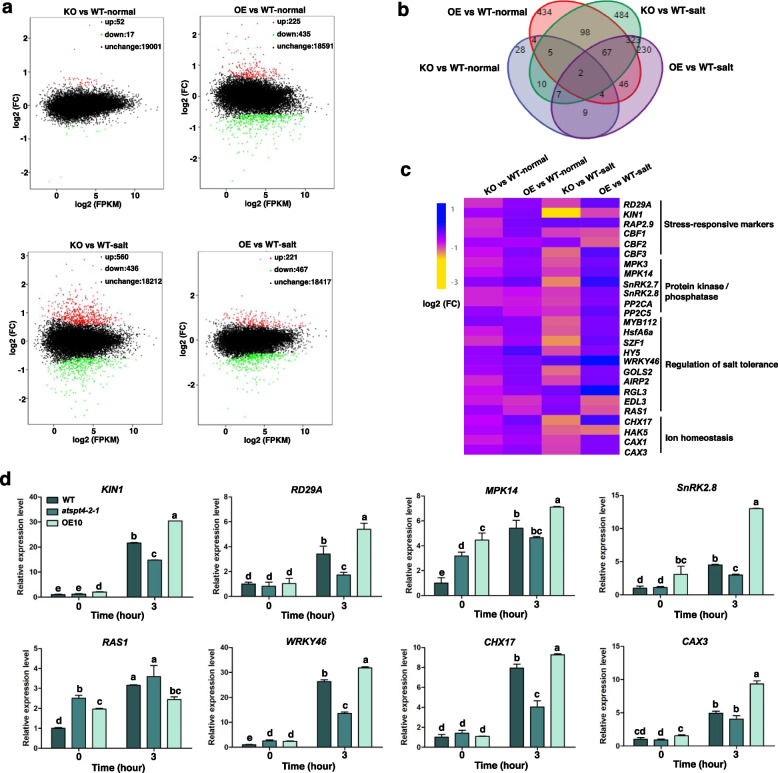
Transcriptomic analyses reveal differentially expressed genes (DEGs) affected by AtSPT4-2. **a** Volcano plot of AtSPT4-2-mediated DEGs. The Y-axis represents the value of log2 (FC), and the X-axis represents the value of log2 (FPKM). Red dots represent upregulated genes, green dots represent downregulated genes, and black dots indicate those without significant changes. **b** Venn diagram of DEGs among the KO vs WT-normal/salt and OE vs WT-normal/salt groups. The number represents the overlapping numbers of DEGs in each of the groups. **c** Hierarchical clustering analyses of DEGs affected by AtSPT4-2. The heatmap represents the transcript abundance of DEGs in different comparison groups. Expression levels for genes are represented as colors ranging from yellow to blue. **d** The transcript levels of stress-responsive genes from **c**. Seven-day-old plants grown on MS medium were transferred to MS medium with 150 mM NaCl for 0, 3 hours, then RNA was isolated from the seedlings for qRT–PCR analyses of the indicated genes. *UBQ5* was used as an internal control. Values are mean ± SD (*n* = 3 replicates, 30 seedlings per replicate). Different letters indicate significant differences by one-way ANOVA (*P* < 0.05)

To explore the regulatory networks of *AtSPT4-2* in the salt stress response, we performed RNA sequencing analysis to identify differentially expressed genes (DEGs) between 7-day-old WT, *SPT4-2* mutant and OE seedlings that were treated with salt for 0 and 3 h. Genes with a fold change in the expression level ≥ 1.5 and a false discovery rate (FDR) ≤ 0.01 were classified as upregulated, and ≤ 1.5 were classified as downregulated. As shown in Fig. 6a, the expression levels of most genes were not significantly altered, and few genes were scattered. Further comparative analysis of DEGs showed that a few up- and downregulated DEGs were functionally characterized in the KO vs WT-normal group, and increased DEGs were exhibited in the KO vs WT-salt group, indicating that loss of *AtSPT4-2* affected the expression of genes involved in the salt stress response. However, Approximate DEGs number was characterized in the OE vs WT-control and OE vs WT-salt groups. In the Venn diagram analysis, a small number of genes were coexpressed in the KO vs WT and OE vs WT groups following normal and salt treatment (Fig. 6b), revealing that AtSPT4-2 may modulate the transcripts of these coexpressed genes. Furthermore, the heatmap analysis shown in Fig. 6c revealed that the transcript levels of the stress-responsive genes *RD29A*, *KIN1*, *RAP2.9*, *CBF1*, *CBF2*, and *CBF3* were upregulated in the OE vs WT group under normal conditions. When subjected to salt treatment, *RD29A*, *RAP2.9*, and *CBF3* were significantly upregulated in the OE vs WT-salt group and downregulated in the KO vs WT-salt group. Moreover, the expression levels of salt-responsive transcription factors, including *MYB112*, *HsfA6a*, *SZF1*, *HY5*, and *WRKY46*, were also higher in the OE line than in the WT under salt stress, while they were lower in the KO line. The transcript abundance of the genes related to ion homeostasis (*CHX17*, *CAX1*, *CAX3*) was also significantly elevated in the OE line. Additionally, the genes encoding protein kinases and phosphatases were affected by AtSPT4-2 (Fig. 6c). Meanwhile, a few DEGs involved in stress response, signal transduction and ion homeostasis were verified in WT, *atspt4-2-1* and OE plants by qRT–PCR. As shown in Fig. 6d, the expression profiles of these genes were largely in agreement with Fig. 6c.

**Fig. 7:**
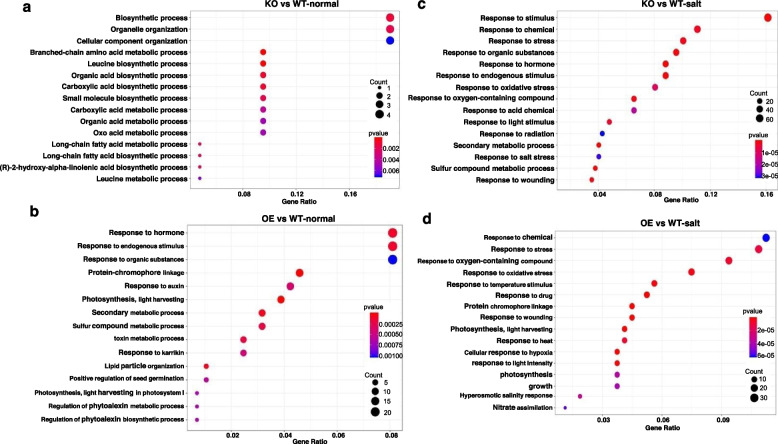
Gene Ontology (GO) analyses of differentially expressed genes affected by AtSPT4-2. **a-d** Functional category of differentially expressed genes. GO enrichment was performed on the set of DEGs identified in KO vs WT-normal (**a**), OE vs WT-normal (**b**), KO vs WT-salt (**c**), and OE vs WT-salt (**d**) groups based on biological processes. The X-axis indicates the ratio of DEGs; the Y-axis indicates the most enriched GO terms

To gain a global view of *AtSPT4-2-*mediated gene expression changes in the salt stress response, we performed Gene Ontology (GO) enrichment analysis using differentially expressed genes. On the basis of the biological processes category, few DEGs in the KO vs WT-normal group were identified as being involved in biosynthetic processes, organelle organization and amino acid metabolic processes (Fig. 7a). Furthermore, a large number of DEGs from the OE vs WT-normal group were assigned to response to hormone and endogenous stimulus, photosynthesis, secondary metabolic process, and toxin metabolic process (Fig. 7b). In the presence of salt stress, the GO categories response to stimulus, stress, hormone and salt stress were predominantly enriched in the KO line (Fig. 7c). Additionally, the genes were highly enriched for the GO terms associated with response to oxidative stress, temperature stimulus, photosynthesis, plant growth and hyperosmotic salinity response in OE vs WT-salt group (Fig. 7d, Table S1). Taken together, these results indicated that AtSPT4-2 mediates salt tolerance by affecting the expression of stress-responsive genes.

## Discussion

In this study, we analysed the role of *AtSPT4-2* in salt tolerance in *Arabidopsis thaliana*. Our genetic analyses with different *AtSPT4-2* genotypes unambiguously show that *AtSPT4-2* plays a positive role in salt tolerance. The overexpression lines exhibited improved salt tolerance at the germination stage and seedling stage as well as plants grown in soil, whereas the knockout mutants showed the opposite phenotype. Our study also revealed that *AtSPT4-2* is induced by salt stress and that the AtSPT4-2 protein appears to always localize in the nucleus and is significantly enhanced by salt stress. This salt-inducible expression and nuclear accumulation of AtSPT4-2 further support that *AtSPT4-2* plays an important role in salt tolerance.

*Thellungiella halophila Salt Tolerance 5* (*ThST5*) is a homolog of *Arabidopsis SPT4-2* gene, which confers salt tolerance in cotton with increased lateral root number and improved fresh root biomass [32, 33]. Further RNA-seq analysis showed that ThST5 regulates the expression of genes encoding antioxidants and salt-responsive transcription factors and maintains ion homeostasis. In this study, our results demonstrated that *AtSPT4-2* positively modulates salt tolerance. Therefore, our study supports the previous evaluation of *ThST5*, which improves salt tolerance in cotton.

Plants have evolved a set of complex mechanisms to cope with salt stress, including salt exclusion, salt elimination, and salt succulence [37]. Maintaining Na^+^/K^+^ homeostasis in the cell cytoplasm by preventing Na^+^ influx and promoting K^+^ uptake is critical for plant survival in the presence of high salinity [38, 39]. The capacity of K^+^ uptake and transport in the roots is correlated with plant salt tolerance. External Na^+^ affected intracellular K^+^ influx by disturbing the ion selectivity of cell membranes [40, 41]. The HAK transporter AtHAK5 is required for K^+^ acquisition to sustain plant growth at low K^+^ under salt stress [42]. The CBL-Interacting Protein Kinase CIPK23 activates HAK5 to mediate high-affinity K^+^ uptake in *Arabidopsis* roots [43]. Recently, it was reported that CHX transporters play an important role in modulating the pH and K^+^ homeostasis of distinct intracellular compartments by altering endomembrane trafficking to adapt to environmental stress [44]. The cation/H^+^ exchanger *AtCHX17* is induced by salt stress and regulates K^+^ acquisition and homeostasis [44, 45]. Loss of *AtCHX17* caused reduced K^+^ accumulation in roots in response to salt stress, thus resulting in K^+^ starvation. Moreover, CHX14 functions as a plasma membrane K-efflux transporter involved in K^+^ redistribution [46]. According to our RNA-seq data, the transcript levels of *HAK5* and *CHX17* are affected by AtSPT4-2. Additionally, the vacuolar H^+^/Ca^2+^ transporters CAX1 and CAX3, which are required for plant growth and ion homeostasis [47], were also upregulated in the OE line but downregulated in the KO line in the presence of salt stress (Fig. 6c). Collectively, our results indicated that AtSPT4-2 might be involved in K^+^ and pH homeostasis regulation in plant cells to confer salt tolerance.

When subjected to salt stress, osmotic balance and ion homeostasis were perturbed. Many stress-responsive genes are induced by abiotic stress and are involved in improving plant tolerance [48]. As shown in Fig. 6c, stress-responsive genes, including *RD29A*, *RAP2.9*, *KIN1*, and *CBF3,* were significantly upregulated in OE plants in response to salt stress. The MAPK signaling pathway-mediated abiotic stress response has been well established in *Arabidopsis* [49, 50]. The MKK9-MPK3/MPK6 cascade plays a vital role in regulating ethylene and camalexin biosynthesis as well as in the salt stress response in *Arabidopsis* [51]. The ABA-activated MAPK cascade, in which MAP 3Ks/MAP 3 K17/MAP 3 K18 activate MKK3 and then stimulate the activity of MPK1/MPK2/MPK7/MPK14, plays an important role in the stress response [52]. SnRK2-mediated signaling is also involved in maintaining osmotic homeostasis, contributing to plant growth [49, 53]. A previous study showed that SnRK2.8 functions as a regulatory factor in the stress response and that overexpression of *SnRK2.8* enhances stress tolerance in *Arabidopsis* [54]. Heterologous overexpression of *AtSnRK2.8* improves resistance to drought and salt stress in *Populus*×*euramericana cv* ‘Nanlin895’ [55]. We found that the transcripts of *MPK3*, *MPK14*, *SnRK2.7*, *SnRK2.8*, *PP2CA*, and *PP2C5* were elevated in OE plants under salt treatment (Fig. 6c), suggesting that AtSPT4-2 modulates the expression of genes associated with signal transduction to enhance plant salt tolerance.

Transcription factors play key regulatory roles in plant salt stress adaptation coupled with sophisticated signal transduction [56, 57]. Several genes encoding salt-responsive TFs were enriched in transcriptomic data, including *MYB112* [58], *HsfA6a* [59], *SZF1* [60], *HY5* [61], and *WRKY46* [62], which were upregulated in OE plants compared to Col-0 plants under salt stress. Moreover, a set of growth-regulating genes under stress, *RGL3* [63], *RAS1* [64], *EDL3* [65], and *GOLS2* [66], were affected by AtSPT4-2 in response to salt stress. ABA, as a plant stress hormone, plays vital functions in adaptive stress processes. Most of these transcription factors or growth-regulating genes modulate stress tolerance dependent on ABA signaling, and the genes associated with ABA signaling, including *SnRK2.7*, *SnRK2.8*, *PP2CA*, and *PP2C5,* were upregulated in OE plants under salt treatment (Fig. 6c), indicating that AtSPT4-2 confers salt resistance possibly by modulating the stress response to ABA, enhancing the induction of stress-responsive genes for ionic and osmotic adjustment.

SPT4, as a transcription elongation factor that interacts with SPT5 via the NGN domain, may promote the elongation of RNAPII-catalyzed mRNA synthesis by reducing the arrest during transcript elongation in *Saccharomyces cerevisiae* [33, 35, 67]. Meanwhile, the SPT4-SPT5 complex is involved in pre-mRNA processing, including capping and splicing [34]. In *Arabidopsis*, SPT5 can directly interact with SPT4, forming the SPT4/SPT5 complex, which is involved in transcript elongation and impacts the expression of auxin-related genes [33]. We speculate that AtSPT4 may facilitate elongation by interacting with the transcript of salt-responsive genes, consequently resulting in elevated transcript levels of these genes, which is consistent with the responsiveness of *AtSPT4-2* expression and AtSPT4-2 protein in the nucleus to salt stress.

## Conclusion

We have demonstrated that *AtSPT4-2* is a positive modulator of salinity tolerance, likely by maintaining intracellular ion homeostasis and increasing the transcript levels of numerous salt-responsive genes, as revealed by our transcriptomic analyses. Therefore, *AtSPT4-2* can be used as a candidate for the improvement of crop salt tolerance.

## Methods

### Plant materials and growth conditions

In the current study, *Arabidopsis thaliana* (Col-0) was used as the wild type (WT), and the transgenic plants were developed in the same background by authors and permitted by University of Science and Technology of China. Two knockout mutants, *atspt4-2-1* and *atspt4-2-2,* were generated by editing the transcription elongation factor (*SPT4-2*) gene in *A. thaliana* through CRISPR/cas-9 using a protocol reported earlier [68], and the knockout (KO) mutants were confirmed through sequencing (Fig. S1a). The *AtSPT4-2* overexpression construct was made by inserting the coding region of *AtSPT4-2* into the vector pCB2004 via the GATEWAY cloning system [69]. The binary vector was transferred into *Agrobacterium tumefaciens* (C58C1) for transformation of *A. thaliana*. Two homozygous overexpression (OE) lines, OE7 and OE10, were selected for further experimental analysis after confirmation of *AtSPT4-2* expression through quantitative RT–PCR using the primers listed in Table S2 (Fig. S1b). Alamin Alfatih conducted the identification of plant materials used in our study. The authors strictly followed all relevant institutional, national, international guidelines and legislation in collecting plants materials and conducting experiments.

The *Arabidopsis thaliana* seeds were sterilized with 10% bleach on constant shaking at 37 °C for 20 minutes followed by rinsing 5 times with sterilized distilled water. The seeds were stratified in the dark for 48 hours at 4 °C. Growth conditions on MS-agar media (Murashige and Skoog) with 1% (w/v) sucrose were maintained at 22 ± 2 °C, 65 ± 5% relative humidity, and a photoperiod of 8 hours dark and 16 hours light.

### Salt tolerance assessment

To assess the seed germination response and survival under salt stress, seeds of all three genotypes, including WT, OE (OE7 and OE10), and knockout mutants (*atspt4-2-1* and *atspt4-2-2*), were horizontally germinated on MS media with or without 150 mM NaCl for 5 days and 220 mM NaCl for 7 days. Germination (the appearance of radicles) and postgermination growth (green cotyledon appearance) were observed every 24 hours.

To assess the response of all three genotypes to salt stress in the soil, the 7-day-old seedlings were transferred to soil pots and allowed to grow for 2 weeks. The 3-week-old plants were irrigated once with or without 250 mM NaCl, allowed to grow for 1 week, reirrigated once with only water and allowed to grow for another 5 days before photographs were taken. The overall morphology and survival rate of the seedlings were assessed.

### RNA extraction and qRT–PCR analysis

TRIzol reagent was used to extract the total RNA of various tissues (Invitrogen, USA). One microgram of the extracted RNA was used to synthesize cDNA with the Prime Script RT reagent kit (Takara, Dalian, China). Reverse transcription polymerase chain reaction (RT–PCR) amplification and quantitative RT–PCR (qRT–PCR) analyses were performed using the cDNA as templates in at least 3 biological replicates for each experiment while using the ubiquitin 5 (*UBQ5*) gene as an internal control. For qRT–PCR detection, the SYBR Premix Ex Taq II kit (Takara) and Applied Biosystems StepOne real-time PCR equipment were employed. Table S2 contains the list of primers used in this study.

### Subcellular localization

The AtSPT4-2-GFP fusion construct was made by inserting the *AtSPT4-2*-coding sequence into the pGWB5 vector [70], and transgenic plants were produced through the floral-dip technique using *Agrobacterium* (C58C1 strain). The green fluorescence of *35S:AtSPT4-2-GFP* transgenic root tissues was observed on a ZEISS 880 confocal laser scanning microscope with an excitation wavelength of 488 nm and an emission wavelength of 525 nm.

### Measurements of antioxidation-associated indexes

The seeds of WT, *atspt4-2-1*, and OE10 were germinated on MS medium without or with 120 mM NaCl for 5 days, and then the whole plants were used for the measurement of proline and malondialdehyde (MDA) contents and antioxidant enzyme activity. Proline content was measured using a Proline Content Determination Kit (CAS: BC0295, Solarbio, Beijing, China). MDA content was measured using a Micro MDA Assay Kit (CAS: BC0025, Solarbio, Beijing, China). Superoxide dismutase (SOD) activity was measured using a SOD Assay Kit (CAS: BC0175, Solarbio, Beijing, China). Peroxidase (POD) activity was measured using a Micro POD Assay Kit (CAS: BC0095, Solarbio, Beijing, China).

### RNA-seq analysis

For RNA-seq analysis, 7-day-old WT, OE10, and *atspt4-2-1 Arabidopsis thaliana* plants were exposed to salt medium containing 150 mM NaCl and then collected after 0 hours and 3 hours of treatment. RNA was isolated using an RNAprep Pure Plant Kit (TIANGEN, Beijing, China). A Bioanalyzer 2100 instrument (Agilent, Santa Clara, CA, USA) (RIN ≥ 7, 28S/18S ≥ 1.5) was used to analyze total RNA integrity. The RNA-seq library was then commercially sequenced by Beijing Biomarker Technology. Differentially expressed genes (DEGs) were characterized by a threshold (absolute value of log2 (fold change) ≥ 1.5, FDR ≤ 0.05) for RNA-seq data. Then, DAVID tools were used to conduct Gene Ontology (GO) term enrichment analysis for each DEG (https://david.ncifcrf.gov/).

### Statistical analysis

SPSS was used for statistical analysis, and one-way ANOVA was used to analyse the data. Values are the mean standard deviation, and statistical significance was set at *P < 0.05*. Different letters indicate a significant difference. The mean values and standard deviations (SDs) were from three biological replicates in each experimental treatment.

### Accession numbers

Sequence data in this study can be found from TAIR: The Arabidopsis Information Resource (www.arabidopsis.org) under the following accession numbers: *AtSPT4-2* (*At5g63670*), *AtRD29A* (*At5G52310*), *AtKIN1* (*At5G15960*), *AtRAP2.9* (*At4G06746*), *AtCBF1* (*At4G25490*), *AtCBF2* (*At4G25470*), *AtCBF3* (*At4G25480*), *AtMPK3* (*At3G45640*), *AtMPK14* (*At4G36450*), *AtMPK14* (*At4G36450*), *AtSnRK2.7* (*At4G40010*), *AtSnRK2.8* (*At1G78290*), *AtPP2CA* (*At3G11410*), *AtPP2C5* (*At2G40180*), *AtMYB112* (*At1G48000*), *AtHsfA6a* (*At5G43840*), *AtSZF1* (*At3G55980*), *AtHY5* (*At5G11260*), *AtWRKY46* (*At2G46400*), *AtGOLS2* (*At1G56600*), *AtAIRP2* (*At5G01520*), *AtRGL3* (*At5G17490*), *AtEDL3* (*At3G63060*), *AtRAS1* (*At1G09950*), *AtCHX17* (*At4G23700*), *AtHAK5* (*At4G13420*), *AtCAX1* (*At2G38170*), *AtCAX3* (*At3G51860*).

## Supplementary Information

**Additional file 1: Fig. S1.** Confirmation of CRISPR/cas9-edited *AtSPT4-2* knockout mutants through sequencing and expression analysis of *AtSPT4-2* overexpression lines through qRT–PCR. a The sequence with mutations in knockout mutant lines compared with the wild-type sequence. Arrows indicate the mutation sites. The deletion in the DNA sequences was designated with “-”. sgRNA, single guide RNA; PAM, protospacer adjacent motif. b Relative expression of *AtSPT4-2* in OE7, OE10, and wild type (WT). Ten-day-old seedlings grown on MS medium were used to quantify the expression of *AtSPT4-2* in transgenic plants along with wild type as a control using qRT–PCR. Ubiquitin was used as an internal reference gene. The values are the mean ± SD (*n* = 3 experiments). **Table S1.** List of overlapping genes related to salt stress between the KO vs WT-control, OE vs WT-control, KO vs WT-salt and OE vs WT-salt groups. **Table S2.** List of primers used in this study.

## Data Availability

The datasets generated during and/or analysed during the current study are available from the corresponding author on reasonable request.

## Abbreviations

Na^+^: Sodium ions
K^+^: Potassium ions
ROS: Reactive oxygen species
TFs: Transcription factors
WT: Wild-type
MDA: Malondialdehyde
SOD: Superoxide dismutase
POD: Peroxidase
DEGs: Differentially expressed genes
GO: Gene Ontology
