# Supplementary material for: Transcription elongation factor AtSPT4-2 positively modulates salt tolerance in Arabidopsis thaliana
Source: BMC Plant Biol. 2023 Jan 23;23:49. doi: 10.1186/s12870-023-04060-x (PMC9869573; doi:10.1186/s12870-023-04060-x)
Supplement: Supplementary file 1 — Additional file 1: Fig. S1. Confirmation of CRISPR/cas9-edited AtSPT4-2 knockout mutants through sequencing and expression analysis of AtSPT4-2 overexpression lines through qRT–PCR. a The sequence with mutations in knockout mutant lines compared with the wild-type sequence. Arrows indicate the mutation sites. The deletion in the DNA sequences was designated with “-”. sgRNA, single guide RNA; PAM, protospacer adjacent motif. b Relative expression of AtSPT4-2 in OE7, OE10, and wild type (WT). Ten-day-old seedlings grown on MS medium were used to quantify the expression of AtSPT4-2 in transgenic plants along with wild type as a control using qRT–PCR. Ubiquitin was used as an internal reference gene. The values are the mean ± SD (n = 3 experiments). Table S1. List of overlapping genes related to salt stress between the KO vs WT-control, OE vs WT-control, KO vs WT-salt and OE vs WT-salt groups. Table S2. List of primers used in this study. [file 12870_2023_4060_MOESM1_ESM.docx]

**Supplementary Materials**

**Transcription elongation factor AtSPT4-2 positively modulates salt tolerance in *Arabidopsis thaliana***

Ayesha Liaqat^1^, Alamin Alfatih^1*^, Sami Ullah Jan^1^, Liangqi Sun^1^, Pingxia Zhao^1*^, Chengbin Xiang^1*^

^1^Division of Life Sciences and Medicine; Division of Molecular & Cell Biophysics, Hefei National Science Center for Physical Sciences at the Microscale; MOE Key Laboratory for Membraneless Organelles and Cellular Dynamics; University of Science and Technology of China, The Innovation Academy of Seed Design, Chinese Academy of Sciences, Hefei, Anhui Province 230027, China.

***Corresponding Authors:**

Chengbin Xiang (Email: [xiangcb@ustc.edu.cn](mailto:xiangcb@ustc.edu.cn), Phone: 0086-55163600429)

Alamin Alfatih (Email: [alfatih@ustc.edu.cn](mailto:alfatih@ustc.edu.cn))

Pingxia Zhao (Email: [zhaopingxia2008@163.com](mailto:zhaopingxia2008@163.com))


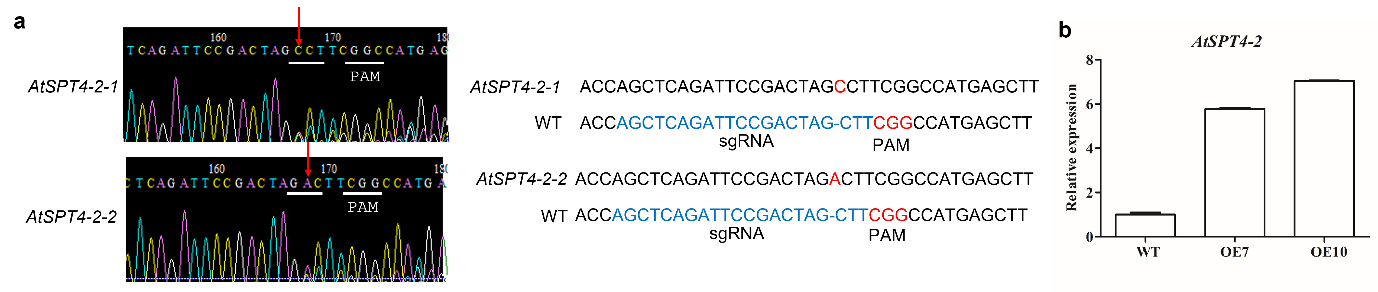


**Fig. S1** Confirmation of CRISPR/cas9-edited *AtSPT4-2* knockout mutants through sequencing and expression analysis of *AtSPT4-2* overexpression lines through qRT‒PCR. **a** The sequence with mutations in knockout mutant lines compared with the wild-type sequence. Arrows indicate the mutation sites. The deletion in the DNA sequences was designated with “-”. sgRNA, single guide RNA; PAM, protospacer adjacent motif. **b** Relative expression of *AtSPT4-2* in OE7, OE10, and wild type (WT). Ten-day-old seedlings grown on MS medium were used to quantify the expression of *AtSPT4-2* in transgenic plants along with wild type as a control using qRT‒PCR. Ubiquitin was used as an internal reference gene. The values are the mean ± SD (n =3 experiments).

**Table S1. List of overlapping genes related to salt stress between the KO vs WT-control, OE vs WT-control, KO vs WT-salt and OE vs WT-salt groups.**

| **Gene ID** | **Annotation** | **Gene Name** | **KO vs WT- control Log2FC** | **OE vs WT- control Log2FC** | **KO vs WT- salt Log2FC** | **OE vs WT- salt Log2FC** |  |
| --- | --- | --- | --- | --- | --- | --- | --- |
| **Stress Responsive Genes** | | | | | | | |
| AT5G52310 | Responsive to Desiccation 29A | RD29A | -0.52039393 | 0.41029292 | -0.658954993 | 0.804301593 |  |
| AT5G15960 | Cold and ABA Inducible Protein KIN1 | KIN1 | 0.2039394 | 0.5209393 | -2.03939393 | -0.69530136 |  |
| AT4G06746 | Related to AP 2 9 | RAP2.9 | -0.5029292 | 0.620393939 | 0.75889041 | 0.762500209 |  |
| AT4G25490 | C-Repeat Binding Factor 1 | CBF1 | -0.4033303 | 0.520339393 | -0.631584669 | -0.76941168 |  |
| AT4G25470 | C-Repeat Binding Factor 2 | CBF2 | 0.520339393 | 0.4033303 | -0.91787415 | 0.820333 |  |
| AT4G25480 | C-Repeat Binding Factor 3 | CBF3 | -0.20393933 | 0.510393848 | -1.026120811 | 0.976100857 |  |
| **Protein kinase/phosphates** | | | | | | |  |
| AT3G45640 | MITOGEN-Activated Protein KINASE 3 | MPK3 | -0.41029292 | 0.323748484 | -0.824739361 | 0.672326635 |  |
| AT4G36450 | MITOGEN-Activated Protein KINASE 14 | MPK14 | -0.2033303 | 0.393939933 | -0.598105163 | 0.82033333 |  |
| AT4G40010 | SNF1-Related Protein KINASE 2.7 | SnRK2.7 | 0.420330303 | 0.761744112 | -1.25867843 | 1.32541771 |  |
| AT1G78290 | SNF1-Related Protein KINASE 2.8 | SnRK2.8 | -0.420293939 | -0.20193933 | -0.589765656 | 0.62033993 |  |
| AT3G11410 | ABA-Hypersensitive Germination 3 | PP2CA | -0.42039393 | -0.320499495 | -0.662351758 | 0.51099393 |  |
| AT2G40180 | Phosphatase 2C5 | PP2C5 | 0.330994944 | -0.24393939 | -0.488828204 | 0.83405885 |  |
| **Transcription Factor** | | | | | | | |
| AT1G48000 | MYB Domain Protein 112 | MYB112 | 0.52030303 | 0.42039393 | -0.93748432 | 0.59039394 |  |
| AT5G43840 | Heat Shock Transcription Factor A6A | HsfA6a | -0.2930944 | 0.420394553 | -0.876758802 | 0.62039393 |  |
| AT3G55980 | Tandem Zinc Finger 11 | SZF1 | -0.530494949 | 0.26404303 | -1.368418867 | 0.76749962 |  |
| AT5G11260 | Basic Leucine Zipper (bZIP) Transcription Factor | HY5 | 0.34030053 | 1.049667815 | -0.750911517 | 0.5203303 |  |
| AT2G46400 | WRKY DNA-Binding Protein 46 | WRKY46 | 0.32039393 | 0.552039394 | 0.893581979 | 1.8203933 |  |
| **Protein** | | | | | | | |
| AT1G56600 | Galactinol Synthase 2 | GOLS2 | 0.20399393 | 0.52399333 | -1.035137193 | 0.58039393 |  |
| AT5G01520 | ABA Insensitive Ring Protein 2 | AIRP2 | -0.429399393 | 0.520393939 | -0.698104174 | 0.62033933 |  |
| AT5G17490 | RGA-Like Protein 3 | RGL3 | -0.20399393 | 0.398289293 | 0.675454495 | 1.82033933 |  |
| AT3G63060 | F-box Protein | EDL3 | -0.32459929 | -0.55303939 | 0.42093939 | -0.9535249 |  |
| AT1G09950 | Response To ABA And Salt 1 | RAS1 | 0.25393933 | -0.420393934 | 0.5532202 | -0.8242494 |  |
| **Ion Homeostasis** | | | | | | | |
| AT4G23700 | Member of Putative Na^+^/H^+^ Antiporter Family | CHX17 | -0.0929293 | 0.694400964 | -1.262402802 | 1.15656769 |  |
| AT4G13420 | High Affinity K^+^ Transporter 5 | HAK5 | 0.09929222 | 0.392200203 | -0.971406481 | -1.1247014 |  |
| AT2G38170 | Cation Exchanger 1 | CAX1 | -0.30939393 | 0.20393933 | -0.685884122 | 0.53099955 |  |
| AT3G51860 | Vacuolar Ca^2+^/H^+^ Transporter | CAX3 | 0.102929393 | 0.420393933 | -0.647501241 | 0.52033003 |  |

**Table S2. List of primers used in this study**

| *AtSPT4-2-OE*  (Forward Primer) | ggggacaagtttgtacaaaaaagcaggctatgtacccatacgatgttccagattacgctATGGCATCAGGAGGTAAAGCC |
| --- | --- |
| *AtSPT4-2-OE*  (Reverse Primer) | ggggaccactttgtacaagaaagctgggtTCATTCCGGACGAGTCTTGAC |
| *AtSPT4-2-GUS*  (Forward Primer) | ggggacaagtttgtacaaaaaagcaggctCAAAATCATTTGCTTTAATCCTTG |
| *AtSPT4-2-GUS*  (Reverse Primer) | ggggaccactttgtacaagaaagctgggtTTTCTTCTAAAGCTGAAAGTGAAC |
| *AtSPT4-2-pGWB5-R* | ggggaccactttgtacaagaaagctgggtgTTCCGGACGAGTCTTGACGAT |
| *AtSPT4-2-qPCR*  (Forward Primer) | ATGGGAAGCGCACCAGCTCA |
| *AtSPT4-2-qPCR*  (Reverse Primer) | TCCATCACAGAGATTATACCATT |
| *AtSPT4-2-CRISPR-R* | GGACAATTCTCGCAACCAGC |
| *AtSPT4-2-CRISPR-LP* | TGACCAAGAAGTCGAGTGTTGT |
| *AtSPT4-2-CRISPR-RP* | CAACCAGCGTCTCTAAACTGTT |
| *AtSPT4-2-GFP*  (Forward Primer) | ATTGGCTCAGATTCCGACTAGCTT |
| *AtSPT4-2-GFP*  (Reverse Primer) | AAACAAGCTAGTCGGAATCTGAGC |
| *KIN1-qPCR*  (Forward Primer) | TCAGAGACCAACAAGAATGCCT |
| *KIN1-qPCR*  (Reverse Primer) | GGCCGGTCTTGTCCTTCACG |
| *MPK14-qPCR*  (Forward Primer) | GCTTATGGTGTGGTTTGTTCTTC |
| *MPK14-qPCR*  (Reverse Primer) | GGAGAGAGACTGAGAGGATTTGA |
| *SnRK2.8-qPCR*  (Forward Primer) | GAGATTCAGTGAAGACGAGGCA |
| *SnRK2.8-qPCR*  (Reverse Primer) | ACTGTTGTCTTTGGTTGCGAATG |
| *RAS1-qPCR*  (Forward Primer) | GAGTGGTGGCGCAGAAGGAG |
| *RAS1-qPCR*  (Reverse Primer) | GGTGAATCAGGTAAAGTAGGGAC |
| *WRKY46-qPCR*  (Forward Primer) | GCTTGCTAACCGATTGATGAACA |
| *WRKY46-qPCR*  (Reverse Primer) | ACTTTCTCTGTGTTCTTCTCCGA |
| *CHX17-qPCR*  (Forward Primer) | CTCTCTCTATCGCTCTCGCC |
| *CHX17-qPCR*  (Reverse Primer) | GCAGCAGCGGACAAAGCAAT |
| *CAX3-qPCR*  (Forward Primer) | ATGGGAGAACAGCACACAACA |
| *CAX3-qPCR*  (Reverse Primer) | CCCTATCAAGCTCAGTCCAA |
